# Severe Hypoglycemia as a Predictor of End-Stage Renal Disease in Type 2 Diabetes: A National Cohort Study

**DOI:** 10.3390/ijerph16050681

**Published:** 2019-02-26

**Authors:** Yu-Li Lee, Shih-Jung Yen, Shyi-Jang Shin, Yi-Chi Huang, Jiun Shiuan He, Kun-Der Lin

**Affiliations:** 1 Department of Internal Medicine, Kaohsiung Municipal Ta-Tung Hospital, Kaohsiung 80145, Taiwan; jangirl1112@yahoo.com.tw (Y.-L.L.); causeyou35@hotmail.com (S.-J.Y.); kmtth8079@gmail.com (J.S.H.); 2 Division of Endocrinology and Metabolism, Department of Internal Medicine, Kaohsiung Medical University Hospital, Kaohsiung Medical University, Kaohsiung 80708, Taiwan; sjshin@kmu.edu.tw; 3 College of Medicine, Kaohsiung Medical University, Kaohsiung 80708, Taiwan; 4 Graduate Institute of Medicine, Kaohsiung Medical University, Kaohsiung 80708, Taiwan; a66225622@gmail.com; 5 Department of Public Health, Kaohsiung Medical University, Kaohsiung 80708, Taiwan

**Keywords:** non-insulin dependent diabetes mellitus, ESRD, hypoglycemia

## Abstract

**Aims:** This study investigated whether there is a link between severe hypoglycemia and progression into end-stage renal disease (ESRD) in patients with type 2 diabetes. **Methods:** Tapping into Taiwan’s Health Insurance Research Database, we identified all type 2 diabetes patients between 1996 and 2013 and identified those diagnosed with a severe hypoglycemia episode during an emergency department visit and those who were not. Controls were then matched 1:1 for age, sex, index year, and medication. **Results:** We identified 468,421 type 2 diabetes patients diagnosed as having severe hypoglycemia in an emergency department visit. Compared with controls, these patients with SH had a higher risk of all-cause mortality (Hazard Ratio (HR), 1.76; 95% confidence interval, 1.61–1.94) and progressed into ESRD within a shorter period of time. Results were similar after controlling for competing risk. **Conclusion:** Severe hypoglycemia is significantly associated with worsening renal dysfunction in patients with type 2 diabetes and hastened progression into ESRD.

## 1. Introduction

About forty percent of type 2 diabetes patients develop chronic kidney disease (CKD) [1], often developing into end-stage renal disease (ESRD). Diabetes experts and clinicians emphasize glucose control in the treatment of diabetes because strict control of blood glucose has been found by many studies to slow the progression of microvascular complications from diabetes mellitus (DM) [2,3,4,5,6]. Severe hypoglycemia, defined as hypoglycemia that requires medical intervention, is the most common and critical cause of adverse events in patients with diabetes [7]. According to one meta-analysis of clinical trials, the relative risk of severe hypoglycemia (SH) is increased by 30% in patients who are placed under intensive glycemic control [8]. Severe hypoglycemia has also been found to be the most common cause of recurrent morbidity people with type 1 diabetes and in people with type 2 diabetes [9], in whom it has also been associated with increased risk of premature death and all-cause cancer [10]. Severe hypoglycemia-related medical costs exceed 100 million dollars annually in the United States, where nearly 100,000 patients with diabetes per year are treated in emergency departments (EDs) [11]. It has also been found to range from 1.4 to 1.6 events per 100 persons per year, and its incidence has been found to be high among elderly patients and patients with CKD—a disease entity associated with increased risk of cardiovascular events related to the associated decrease in glomerular filtration rates [11,12,13]. However, to the best of our knowledge, no study has investigated the possible link between severe hypoglycemia and progression into ESRD in Taiwan. This longitudinal observational case-cohort study collected data from Taiwan’s universal health insurance claims database for 1996 to 2013 to study the association between emergency department diagnosed SH events in type 2 diabetes patients and subsequent development of CKD.

## 2. Subjects and Methods 

In this longitudinal observational cohort study, we collected medical claims and recipient data submitted to Taiwan’s National Health Insurance program from 1996 to 2013. The National Health Insurance Database (NHIRD) contains enrollment information, outpatient and inpatient diagnoses, and claims for medical services and medications, and catastrophic illness registration data. This database was used to identify patients diagnosed as having diabetes and their comorbid conditions. All NHIRD data were obtained from Taiwan’s National Health Research Institutes, the organization responsible for maintaining the database and making it available to researchers. The Institutional Review Board of Kaohsiung Municipal Ta-Tung Hospital (KMTTHIRB-E(I)-20160123) approved this study.

Diabetes mellitus was defined in patients if they had a primary diabetes diagnosis (ICD-9-CM code 250) and had received prescriptions for an oral antidiabetic drug for ≥84 days during the time period 2000 to 2008. We included all patients with type 2 diabetes who had complete enrollment information. Severe hypoglycemia was identified in DM patients if they had received any SH diagnosis (ICD-9-CM codes 251.0, 251.1, and 251.2) listed in Taiwan’s Emergency Department (ED) Database from 2000 to 2008. Severe hypoglycemia patients were defined by a hypoglycemia event and were sent to an ED by ambulance during 2000–2008. Severe hypoglycemia index date was defined as date of first diagnosis of SH. Diabetic controls, who did not have SH, were randomly assigned index dates based on a dynamic frequency distribution of time exposed to diabetes in the SH group. We excluded all patients diagnosed with cancer, ESRD, or diabetes with renal manifestations before the index date and patients who did not submit claims for prescription of an oral antidiabetic drug after the index date. In total, we identified 277,433 DM patients with no SH and 4019 DM patients with SH. To avoid potential confounding from selection bias, we used propensity score matching methods to match those with and without SH 1:1 by sex, age, DM duration, and Charlson comorbidity index (CCI) scores. After matching, 4017 DM patients with SH were paired with 4017 DM patients without SH and entered into our analysis.

The main endpoints of this study were ESRD and all-cause mortality. End-stage renal disease was identified as a diagnosis of ESRD coded using an ICD-9 CM code and enrollment into the national catastrophic illness registry database. All-cause mortality was identified by death events recorded in the National Health Insurance Enrollment Database. Baseline characteristics that may have affected outcomes were included as control variables. Demographic covariates were age and sex. Charlson comorbidity index scores within one year before index date were used to measure patients’ baseline comorbidities, which included hypertension (ICD-9-CM codes 401–405) and hyperlipidemia (ICD-9-CM code 272). Prescriptions for insulin (ATC code = A10AAxx), metformin (ATC code = A10BA02), sulfonylurea (ATC code = A10BBxx), ACTOS (ATC code = A10BG03), and glucobay (ATC code = A10BF01) were examined but prescriptions for DPP4 inhibitor were not because this drug did not appear on Taiwan’s market until 2013. Dose-duration-day (DDD) was cumulatively calculated as the number of days that the antidiabetic drugs were prescribed from the index date until the date of ESRD diagnosis, death, or the end of follow-up date.

### Statistical Analysis

The χ^2^ test was used to evaluate differences in sex, age, DM duration, CCI score, and antidiabetic drug prescription between patients with and without SH; however, mean age, mean DM duration, and mean CCI score were examined using the independent sample *t*-test. Univariate and multivariate Cox proportional hazards regressions were performed to measure the risks of ESRD incidence and all-cause mortality. Hazard ratios (HRs) and 95% confidence intervals (CIs) were determined. Potential confounding variables were sex, age, DM duration, CCI score, DDD of antidiabetic drugs, and comorbidity. These potential confounding variables were controlled for in our multivariate models. We used Kaplan–Meier survival curves to estimate the effects of time factors on the death and ESRD incidence and cumulative survival rate. We used the log–rank statistic test to assess differences between the SH and non-SH groups. All statistical operations were performed using SAS version 9.3 (SAS institute, Cary, NC, USA) and Stata version SE 11. Two-tailed *p*-values lower than 0.05 were considered significant.

## 3. Results

**Figure 1:**
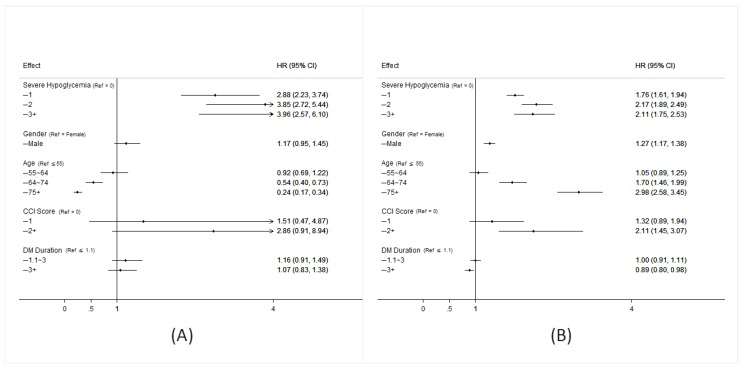
Hazard ratio for all-cause mortality (**A**) and of incidence of ESRD (**B**) in patients with type 2 diabetes among each group. (A crude model was calculated by entering one variable at a time. Cox regression of each factor adjusted by hypoglycemia, age, gender, Charlson comorbidity index (CCI), DM duration. HR = hazard ratio).

**Figure 2:**
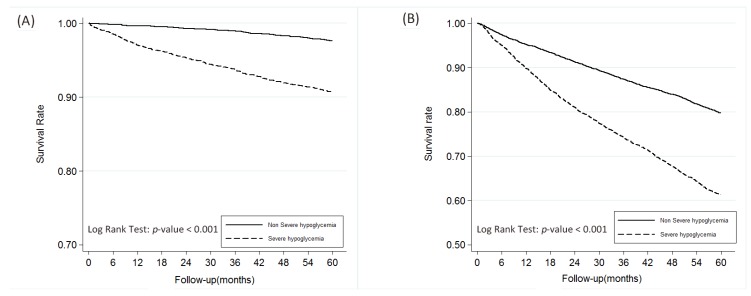
The overall survival rate of patients with between non-severe hypoglycemia and severe hypoglycemia groups (**A**) and end-stage renal disease-free survival rate (**B**).

As can be seen in Table 1, which presents the characteristics of the propensity score matched patients with and without severe hypoglycemia, there were no significant differences in the two cohorts with regard to age, sex, DM duration, insurance ranges, or CCI scores. There were, however, significant differences in medications used by the two groups. The SH cohort used more insulin and glucobay, while the cohort that did not develop SH used more sulfonylurea, metformin, and Actos. As shown in Figure 1A, patients diagnosed with SH, those who had a higher incidence of SH events, males, those over 65 years old and those with CCI scores > 2 had higher mortality rates. A higher risk of ESRD was associated with a diagnosis of severe hypoglycemia, the higher the incidence, the greater the risk. Those >65 years old had lower risk of ESRD due to competing risk of mortality (Figure 1B). As can been seen in Figure 2A, the SH cohort had a lower survival rate. As can be seen in Figure 2B, diabetic patients with SH were found to progress more rapidly into ESRD those without SH.

## 4. Discussion

This study found a strong correlation between severe hypoglycemic and quicker progression into ESRD among people with type 2 diabetes, indicating the occurrence of these events predicted and may have aggravated kidney failure in this population in Taiwan. Numerous studies have reported that hypoglycemia can cause vascular damage in the heart, brain and nerve, but few studies on SH focus on its influence on CKD and ESRD. To the best of our knowledge, no previous study has reported the association between SH and hastened progression to ESRD in diabetes patients.

The current nationwide cohort study found a strong association between SH and mortality and CKD. Similarly, the ACCORD and ADVANCE trials, reporting results of their post-hoc analyses, indicated a strong correlation between hypoglycemia and mortality [8,11,14,15,16]. A retrospective cohort study by Zhao et al. [17] reported higher incidence rates of microvascular complications in veterans with type 2 diabetes than those without type 2 diabetes. Our study found type 2 diabetes patients who had had SH events progressed more rapidly into ESRD than diabetic patients who had not had SH events.

It is common in our practice to find that patients who have repeated hypoglycemic events often lack knowledge about hypoglycemia and its effects. Hypoglycemia can affect endothelial function, inflammatory cytokine secretion, fibrinolysis, and coagulation, which increase vascular morbidity and mortality. It also stimulates the release of catecholamines, further affecting health. All such responses can lead to inflammation- and vasoconstriction-induced vascular changes [18,19], some resulting in long-term vascular damage. This study found the higher the SH incidence, the higher the ESRD risk.

The negative affect that severe hypoglycemia has on kidney function may occur through acute kidney injury. Diabetic nephropathy has been associated with tissue inflammation, including increases in cytokine of TGF-β (CTGF) and tumor necrosis factor (TNF)α [20]. Acute kidney injury has been found to damage adhesion endothelium and cause maladaptive repair of kidney cells [21]. Pereira BJ et al. [22] found that patients who had had acute kidney injuries had decreased glomerular filtration rates at follow up. Previously, we also reported that patients who had had an SH event also had decreased glomerular filtration rates at 8-months follow-up, the most vulnerable patients being those with higher baseline blood creatinine values [23]. Hypoglycemia might induce a greater need for oxygen which could tax the system leading to endothelial dysfunction with failure to vasodilate and vessel injury. Hypoglycemia also induces the production of several inflammatory markers, including interleukin(lL)-6, C-reactive protein, TNFα, IL-8, and endothelin-1, which can cause endothelial injury. After a hypoglycemia event, vascular endothelial growth factor has been found to be increased locally and in circulation [19]. Interleukin-1 also increases the severity of hypoglycemia, creating a positive feedback cycle [19]. Considered together, we think that severe hypoglycemia may hasten ESRD by speeding up the progression of diabetic nephropathy, though further research is needed to explore this hypothesis.

Authors of the ADVANCE trial report suggested that the cause of microvascular disease was more likely to be longer diabetic duration and increased age than a direct effect of hypoglycemia [10]. Our study found severe hypoglycemia to be an independent risk factor for ESRD, after adjusting these two covariates. It could also be argued that other underlying diseases might also be more of a cause of kidney injury than severe hypoglycemia. However, in this study, patients with and without SH had higher CCI scores around two. We found SH to remain an independent risk factor of ESRD, after adjusting for age, DM duration, and CCI score. It might also be argued that progression of CKD in our patients was more a result of diabetic nephropathy than of SH. However, as can been seen in curves Figure 2B, changes in the curve for the patients with type 2 diabetes who experienced SH in time to ESRD. Furthermore, it might also be argued that T2DM patients with CKD are at higher risk of hypoglycemia than those without the comorbidity [24]. Cheng et al. [25] also reported patients with diabetes were more likely to develop ischemic heart disease, chronic kidney disease, and stroke than other diabetic complications including myocardial infarction, chronic heart failure, and ESRD in Asian population. In this study, SH denoted a risk factor prior to developing mortality and ESRD. However, our groups were matched for age, sex, diabetes duration, insurance range, CCI score, and medications (i.e., insulin, sulfonylurea, metformin, and glucobay) for the same observation period. This close matching increases the validity of our finding that hypoglycemia directly hastens progression into ESRD in patients with diabetes.

An ADA workgroup [26] and an ADA consensus conference on diabetic kidney disease [27] have suggested that clinicians should exercise precaution when selecting and dosing SU and insulin for patients with diabetes and CKD. All DDP-4 inhibitors may be used in elderly patients or patients with CKD who are at low risk of hypoglycemia. The OADs have been thought to lead to improvement in SH, but one study of trends in drug utilization based on 2006 to 2013 data of people with type 2 diabetes found that the overall rate of SH was largely unchanged when clinicians prescribed glucose-lowering medications and decreased sulfonylureas [28]. Although our study lacked data on DPP4i, which is used more commonly today, our results contribute to the body of evidence suggesting that SH has serious adverse health effects and more attention should be devoted to its prevention.

This study has several limitations. One limitation is that our definitions were based on primary exposure and outcomes based on ICD-9-CM codes listed on insurance claims, and there is a possibility of misclassification. However, the accuracy of these codes were validated. What little misclassification there may have been would probably not cause a difference in our comparisons of the two cohorts. Another limitation is that study is a retrospective observational study and causal relationships cannot be determined with confidence. Still another limitation is that we had no access to biochemical data, which were not available in the health insurance claims database. The correlation between SH and outcomes is not linearly correlated and is perhaps due to the differences in the severity of hypoglycemia. However, we could not subgroup our SH patients by hypoglycemia severity and could not further analyze this in our database.

## 5. Conclusions

In conclusion, this study shows that SH adversely affects patients with type 2 diabetes not only by increasing the risks of MI, stroke, and mortality, as has already been established, but also by increasing the risks of microvascular disease and ESRD. Physicians should exercise caution when prescribing glucose control medications to people with type 2 diabetes, especially to those who have already had a severe hypoglycemic event. Physicians should aim to control glucose as well as minimize SH risk in order to prevent or delay progression into ESRD in this population.

## Figures and Tables

**Table 1:** Characteristics of patients before and after propensity score matching between severe hypoglycemia and control groups.

	Severe Hypoglycemia	Non-Severe Hypoglycemia	*p*-Value
	Mean ± SD/(*N*, %)	Mean ± SD/(*N*, %)	
*N*	4017	4017	
Sex ^a^ (*N*, %)	1846 (45.95%)	1846 (45.95%)	1.000
Male	2171 (54.05%)	2171 (54.05%)	
Female	67.92 (±12.87)	67.52 (±12.20)	0.154
Age (Mean ± SD)			
Age (*N*, %) ^a^	660 (16.43%)	656 (16.33%)	0.999
<55 years old	762 (18.97%)	761 (18.94%)	
55–64 years old	1237 (30.79%)	1242 (30.92%)	
65–74 years old	1358 (33.81%)	1358 (33.81%)	
75+ years old	2.33 (±1.86)	2.12 (±1.62)	<0.0001
DM duration (Mean ± SD)			
DM duration ^a^ (*N*, %)	1321 (32.89%)	1327 (33.03%)	
Less than 1 year	1369 (34.08%)	1366 (34.01%)	
1–3 years	1327 (33.03%)	1324 (32.96%)	
More than 4 years	2.99 (±1.81)	2.99 (±1.82)	0.946
CCI scores ^a^ (Mean ± SD)			
CCI scores (*N*, %)	85 (2.12%)	87 (2.17%)	0.988
0	850 (21.16%)	850 (21.16%)	
1	3082 (76.72%)	3080 (76.67%)	
2+			
Metformin use ^b^	1364 (33.96%)	970 (24.15%)	<0.0001
No	2653 (66.04%)	3047 (75.85%)	
Yes			
Sulfonylureas use ^b^	1316 (32.76%)	840 (20.91%)	<0.0001
No	2701 (67.24%)	3177 (79.09%)	
Yes			
Insulin use ^b^	2887 (71.87%)	3503 (87.20%)	<0.0001
No	1130 (28.13%)	514 (12.80%)	
Yes			
ACTOS use ^b^	3679 (91.59%)	3614 (89.97%)	0.012
No	338 (8.41%)	403 (10.03%)	
Yes			
Glucobay use ^b^	3221 (80.18%)	3298 (82.10%)	0.028
No	796 (19.82%)	719 (17.90%)	

Abbreviations: CCI, Deyo–Charlson comorbidity index; PSM, propensity score matching; ^a^ These variables were used in the PSM. ^b^ Anti-diabetes drug use was defined if patients had been prescribed the selected medications between the index date and the study end date (31 December 2013), follow-up five years, end-stage renal disease (ESRD) occurrence, or death date, whichever came first; patients could use more than one drug.
